# Microwave field mapping for EPR-on-a-chip experiments

**DOI:** 10.1126/sciadv.ado5467

**Published:** 2024-08-16

**Authors:** Silvio Künstner, Joseph E. McPeak, Anh Chu, Michal Kern, Markus Wick, Klaus-Peter Dinse, Jens Anders, Boris Naydenov, Klaus Lips

**Affiliations:** ^1^ Helmholtz-Zentrum Berlin für Materialien und Energie GmbH, Hahn-Meitner-Platz 1, 14109 Berlin, Germany.; ^2^ Institute of Smart Sensors, Universität Stuttgart, 70569 Stuttgart, Germany.; ^3^ Institute for Microelectronics Stuttgart (IMS CHIPS), Allmandring 30a, 70569 Stuttgart, Germany.; ^4^ Center for Integrated Quantum Science and Technology (IQST), Stuttgart and Ulm, Germany.; ^5^ Berlin Joint EPR Laboratory, Fachbereich Physik, Freie Universität Berlin, 14195 Berlin, Germany.

## Abstract

Electron paramagnetic resonance–on-a-chip (EPRoC) devices use small voltage-controlled oscillators (VCOs) for both the excitation and detection of the EPR signal, allowing access to unique sample environments by lifting the restrictions imposed by resonator-based EPR techniques. EPRoC devices have been successfully used at multiple frequencies (7 to 360 gigahertz) and have demonstrated their utility in producing high-resolution spectra in a variety of spin centers. To enable quantitative measurements using EPRoC devices, the spatial distribution of the *B*_1_ field produced by the VCOs must be known. As an example, the field distribution of a 12-coil VCO array EPRoC operating at 14 gigahertz is described in this study. The frequency modulation–recorded EPR spectra of a “point”-like and a thin-film sample were investigated while varying the position of both samples in three directions. The results were compared to COMSOL simulations of the *B*_1_-field intensity. The EPRoC array sensitive volume was determined to be ~19 nanoliters. Implications for possible EPR applications are discussed.

## Teaser

High-resolution spatial mapping of the microwave field enables quantitative EPR on chip measurements.

## INTRODUCTION

Electron paramagnetic resonance (EPR) spectroscopy is a widely used, powerful, and noninvasive spectroscopic technique sensitive to and selective for paramagnetic species. It is used in a variety of fields such as physics, chemistry, biology, materials science, life science, and medicine. EPR is commonly applied for quality control and chemical analyses, the identification and characterization of radicals (*1*), paramagnetic defects (*2*–*4*), and spin-dependent processes in semiconductors and devices (*5*, *6*), transition metal ion states in biological samples, and for the assignment of the electronic and atomic structure of paramagnetic states during chemical reactions (*7*). Not only can EPR yield qualitative information about the paramagnetic species, but it can also be used to quantify the concentration of specific paramagnetic centers in the specimen (*8*). Most commonly, quantitative EPR is used for radiation dosimetry (*9*–*12*), environmental research (*13*–*16*), archaeological and geological dating (*17*–*19*), food analysis and control (*20*–*24*), medical diagnostics (*25*–*30*), and materials science (*3*, *4*, *31*–*36*).

**Fig. 1.**
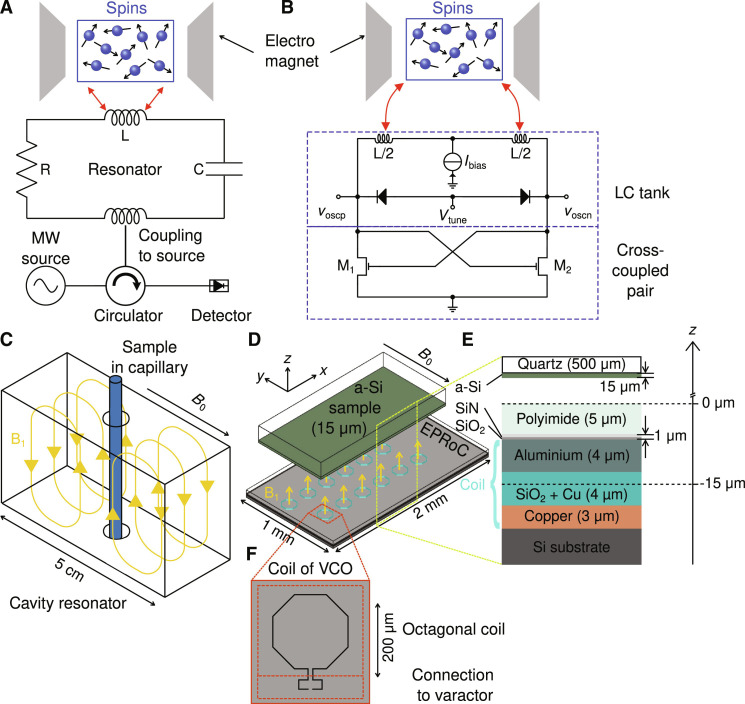
Comparison of resonator-based and VCO-based EPR detection showing the differences in the working principles. (**A**) Schematic representation of a simplified resonator-based CW reflection-type EPR spectrometer and equivalent circuit diagram of an RLC-resonator interacting with the spins in the sample. (**B**) Equivalent circuit diagram of a VCO-based EPR spectrometer interacting with spins in the sample. The EPR signal may be detected as a frequency change (dispersion-like) in the VCO when fulfilling the resonance condition. Adapted from (*87*). (**C**) Sketch of a TE-102 MW cavity resonator commonly used at X-band. The direction of **B**_**0**_ ⊥ **B**_**1**_ is indicated by the arrow. (**D**) Sketch of a 12-coil VCO array chip with a thin-film test sample positioned above the chip. Each coil produces a **B**_**1**_ field as indicated. For clarity only, the **B**_**1**_ component perpendicular to the plane is indicated. A thin film of a-Si (green) on a glass substrate (opaque) is used as a sample that is placed above the coils with Si pointing toward the array. (**E**) Layer structure of the coil on the chip. The origin of the *z* axis is on the surface of the chip, i.e., on the polyimide layer, designated as *z* = 0. The “center” of the coil is approx. at *z* = −15 μm. The *z* axis is not shown to scale. (**F**) Top view of the shape of single coil of a VCO. For further details, see text.

For routine quantitative EPR measurements, commercial continuous wave (CW) EPR spectrometers are used. A simplified sketch of the components of such a spectrometer is displayed in Fig. 1A. The setup typically uses microwave (MW) bridges containing an MW source and detector in combination with an MW cavity resonator (volume ~ cubic centimeter, see Fig. 1C) with a large quality factor, *Q*, to enhance the signal-to-noise ratio (SNR). The resonator couples the MW magnetic field component, *B*_1_, to the magnetic susceptibility, χ, of the paramagnetic sample (Fig. 1C). The external magnetic field, *B*_0_, is swept using an electromagnet while keeping the MW frequency constant to obtain an EPR spectrum. To quantify the paramagnetic species, the spectrometers are commonly calibrated using samples with a known number of spins or spin density, e.g., nitroxide solutions with known molar concentrations.

In the ideal case, the exciting *B*_1_ field should be uniformly distributed over the entire sample; however, in practice, the sample may extend beyond the effective *B*_1_ field or may occupy only a small fraction of the total *B*_1_ field, greatly complicating a quantitative analysis. For a successful quantitation of an extended sample, either a calibration sample of the same extent with a known number of spins is necessary to minimize the effect of inhomogeneous *B*_1_ fields or the spatial distribution of the *B*_1_ field in the resonator must be determined experimentally to correct for different signal amplitudes originating from inhomogeneities in the *B*_1_ field distribution over the sample volume. Such a calibration sample may, however, not always be available or may lead to an erroneous quantification if the geometry and paramagnetic species are not exactly the same. Furthermore, only the total number of spins may be obtained. Using the *B*_1_ field distribution for quantitation does not suffer from such issues so that it is advantageous specifically for applications where the number of spins per unit volume is the figure of merit, such as in fluid solution. Usually, the spatial distribution is determined by comparing the EPR signal of a point sample relative to the sample position within the cavity.

Because cavity resonators are optimized for relatively large samples (100 to 1000 μl), the sensitivity for volume- or mass-limited samples is far from optimal because of the inherently small filling factor, η, of most resonators (*37*), which is defined as

$$\eta =\int_{V_{s}}B_{1r}^{2}dV/\int_{V}B_{1}^{2}dV$$ (1)

where *B*_1_ is the MW magnetic field in the resonator volume, *V*, and *B*_1r_ is one of the rotational components (clockwise or counterclockwise) of the linear *B*_1_ field perpendicular to *B*_0_ within the sample volume, *V*_s_. Dielectric or ferroelectric inserts in standard EPR cavities, small solenoidal coils, or other specialized resonant structures (*38*–*42*) improve sensitivity for samples with volumes around 1 μl. For submicroliter samples such as protein crystals or thin films, dedicated three-dimensional (3D) (*43*–*48*) and planar (2D) microresonators (*46*, *48*–*50*) have been developed, which improve the sensitivity for these kinds of samples due to the increased filling factor. While 3D microresonators have a relative good *B*_1_ homogeneity such as loop-gap resonators (*43*–*45*) or microhelices (*47*), planar microresonators exhibit large *B*_1_ gradients over the sensitive volume, resulting in highly inhomogeneous *B*_1_ distributions in the sample, which poses a problem for pulsed operation and quantitative EPR. For the former, pulse shaping can compensate for some degree of *B*_1_ inhomogeneity enabling uniform excitation (*51*, *52*), while for the latter, detailed knowledge about the *B*_1_ distribution is necessary to obtain quantitative information about the sample (*8*). The usage of microresonators for routine quantitative EPR experiments is limited due primarily to the necessity for an MW bridge and also because of the inherently complicated coupling procedure to the MW source (*53*). In addition, the investigation of polar samples such as proteins and cells in aqueous solution using microresonators is problematic because of the nonresonant absorption of MWs due to the interaction with the electric field (*E*_1_), of the MW, which is not completely separated from *B*_1_.

All of these limitations, except *B*_1_ inhomogeneity, may be removed simultaneously by using miniaturized EPR spectrometers fully integrated on a single microchip such as those developed recently (*54*–*58*). All of these devices integrate a conventional MW bridge, or variants thereof, into either a single integrated circuit (*56*, *58*), a fixed-frequency oscillator–based design (*54*, *55*, *59*), or a voltage-controlled oscillator (VCO)–based design (*57*, *60*, *61*). In the VCO-based approach shown in Fig. 1 (B and E), the so-called EPR-on-a-chip (EPRoC) consists of a planar, typically single-turn inductor or coil with a diameter of several hundred micrometers embedded in a VCO serving as both the MW source and detector simultaneously. This design is based on an idea first proposed in the 1950s in nuclear magnetic resonance (NMR) spectroscopy (*62*). In this approach, the MW power depends on the VCO bias current. Using VCOs as MW sources enables the recording of frequency-swept EPR spectra, which then enables the usage of permanent magnets for smaller battery-operated in-field spectrometers, as shown recently (*57*, *63*, *64*). EPRoC devices have been successfully used at multiple frequencies (7 to 263 GHz) and have demonstrated their utility in producing high-resolution spectra in a variety of spin centers such as copper, manganese, chromium, vanadium, nitroxides, and other small organic radicals at temperatures between 1.4 K and room temperature (*55*, *59*, *65*–*68*). One notable advantage of EPRoC compared to conventional microresonators is the possibility to manufacture arrays of injection-locked VCOs, so-called EPRoC arrays (*69*), as shown in Fig. 1B, which exhibit improved sensitivity relative to a single EPRoC due to lower phase noise (*60*, *68*). In EPRoC arrays, a planar array of coils constitutes the sensor instead of a single coil. Hence, single-coil EPRoC and EPRoC arrays seem ideally suited for the quantitative investigation of both submicroliter-volume samples in which the sample is much smaller than the coil size and thin film samples that cover multiple coils for which the lateral dimensions of the sample are much larger than a single coil. To obtain quantitative results with such samples using the EPRoC array, it is necessary to investigate certain signal properties of EPRoC arrays, such as the dependence of the EPR signal on the sample position within a single coil and probing the entire array in all spatial directions, which goes hand in hand with the sensitive volume that is probed by the EPRoC. The sensitive volume is especially relevant to determine the spin concentration in fluid solutions. In addition, the behavior of the EPRoC array on an inhomogeneously distributed sample must be determined.

Herein, we present experimental data and simulation of the *B*_1_ field distribution in a 12-VCO array EPRoC sensor investigated using a “point”-like sample of α,γ-bisdiphenylene-β-phenylallyl (BDPA) and a homogeneous thin-film sample of amorphous silicon on a glass substrate (a-Si, 15 μm thin) to determine the sensitive volume of a single coil as well as the complete array, the dependence of the EPRoC signal on the sample position, and the dependence of the EPRoC signal during partial coverage of the array coils. With these calibration experiments, the capabilities of EPRoC sensors for quantitative EPR measurements for in-the-field deployment are demonstrated.

In Fig. 1, a comparison of EPR techniques (Fig. 1, A and B) and their detection principles (Fig. 1, D and E) is shown. Commercial EPR spectrometers commonly use high *Q* MW cavity resonators as shown in Fig. 1A to improve the SNR of the EPR signal. The sample usually resides in a capillary tube with a diameter of a few millimeters (X-band, ~9 GHz), which is inserted in the center of the resonator. The size of these resonators, such as the commonly used TE-102, is in the range of the wavelength of the MW such that a standing wave is formed within the cavity. In a well-designed cavity resonator, the electric (*E*_1_) and magnetic (*B*_1_) fields of the MW are spatially separated to maximize the resonant absorption of the sample via *B*_1_ and to reduce the nonresonant absorption via *E*_1_. The condition that the MW frequency and the spin resonance frequency must coincide imposes a restriction on the dimensions of the resonator. Hence, resonators used at X-band frequencies have volumes of several cubic centimeters, which are much larger than the typical sample size of tens of cubic millimeters. As shown in Fig. 1C, the MW source (usually a Gunn diode) produces the MW, which is fed into the resonator through a circulator, ensuring a unilateral coupling of the resonator and with that the spin system to the MW source. From the resonator, the MWs pass through the circulator again and are recorded using a diode detector. The equivalent circuit diagram of a cavity resonator is an RLC resonator, where the inductance, *L*, and the capacitance, *C*, determine the resonance frequency. In this picture, the sample is placed in the inductive loop of the RLC resonator described by *L*. One drawback of resonator-based spectrometers is the limited bandwidth such that to obtain an EPR spectrum, the magnetic field must be swept, which necessitates the use of an electromagnet.

A sketch of an EPRoC sensor is shown in Fig. 1D, which shows an injection-locked 12-coil VCO array device. The equivalent circuit diagram of a single VCO is shown in Fig. 1B. Compared to resonator-based designs, the VCO constitutes both the MW source and detector simultaneously, removing the need for additional MW hardware (MW source, circulator, and diode detector). The VCO can be described as an LC tank consisting of an inductance *L* and a varactor *C* combined with a cross-coupled transistor pair (*70*) with a negative small signal resistance that replenishes the energy dissipated in the LC tank. Here, the coil of the VCO both produces the *B*_1_ field and detects the change in sample magnetization. The bias current, *I*_bias_, applied to each VCO in parallel determines the magnitude of *B*_1_. A certain minimum bias current is needed to obtain stable (MW) oscillations, i.e., there is always a certain minimum *B*_1_, which cannot be further reduced. The coil as depicted in Fig. 1F consists of two parts: an octagonal coil with a diameter of 200 μm (top) and the legs connecting it to the varactor of the LC tank. This detection principle is fundamentally different from the resonator-based approach in the sense that the VCO and the spin system are bidirectionally coupled. Thus, when the spin system absorbs and disperses the exciting MWs, the amplitude and frequency of the VCO change. Because of the constant delivery of current to the coil, an absorption-like amplitude-modulated (AM) EPR signal (*59*, *71*, *72*) can be detected as the change in oscillation amplitude of the VCO, while a dispersion-like frequency-modulated (FM) EPR signal is observed via the change in frequency of the VCO (*54*, *55*, *73*).

In the experiments reported herein, only the frequency-sensitive detection mode of EPRoC is used. The FM signal, Δω_osc,spin_, may be described by Eq. 2 (*73*)

$$\Delta \omega_{\text{osc},\text{spin}}\approx -\frac{1}{2}\;\omega_{\text{osc},0}\frac{L_{\text{spin}}}{L_{0}}$$ (2)

in which ω_osc,0_ = ω_osc,spin_ (*L*_spin_ = 0) is the oscillation frequency in the absence of coupling to spins (EPR), $L_{0}=\frac{1}{\mu_{0}}\int_{V}B_{1u}^{2}dV=\text{const}$. The inductance due to the EPR, *L*_spin_, may be calculated from a magnetic energy–based approached in combination with the steady-state solution of Bloch’s equations as follows

$$L_{\text{spin}}=\gamma T_{2}^{2}\int_{V_{S}}\frac{M_{0}\cdot \omega_{\text{osc},0}-\omega_{L}\cdot B_{1u}^{2}}{1+{\omega_{\text{osc},0}-\omega_{L}}^{2}T_{2}^{2}+\gamma^{2}B_{1}^{2}T_{1}T_{2}}dV$$ (3)

where γ = g μ_B_ / *ħ* is the gyromagnetic ratio, ω_L_ = −γ*B*_0_ is the Larmor frequency, *V*_s_ is the sample volume, *M*_0_ = χ_0_ *B*_0_ / μ is the steady-state sample magnetization, *B*_1_ is the magnitude of the MW magnetic field, *B*_1u_ is the unitary magnetic field of the coil, i.e., the magnetic field produced by 1-A current in the coil, and *T*_1_ and *T*_2_ are the longitudinal and transverse relaxation times of the sample, respectively. Here, the inductance due to the EPR, *L*_spin_, may be interpreted as the change in total inductance of an inductor containing an EPR-active sample. Equation 2 is a first-order approximation for the EPRoC signal and disregards the bidirectional coupling of the EPRoC described above.

Within the EPRoC 12-coil array, the VCOs present on the chip (*N* = 12) are injection-locked through coupling resistors. The interconnection of the VCOs is such that *B*_1_ is in phase, i.e., the direction of *B*_1_ at each point in time is the same within each coil similar to the design in reference (*60*). In addition, the phase noise of the total oscillation frequency is lowered by $\sqrt{N}$ . The reduction of the phase noise of the VCO array may be intuitively explained by a correction force on the joint oscillation phase that the other *N*-1 VCOs exert whenever the phase of a single VCO deviates from its nominal value. In total, this may lead to an improved sensitivity by $\sqrt{N}$ for the FM signal of the array compared to a single VCO. The AM detection does not gain from the lowering of the phase noise, while the FM detection does. Hence, in this work, only the FM-detected signal is investigated.

The frequency of the VCOs may be adjusted by changing the voltage applied to the varactors, the so-called tuning voltage, *V*_tune_, which is applied in parallel to all VCOs, allowing the use of a permanent magnet instead of an electromagnet, which is, however, not used in this work. This oscillator-based design allows for the construction of completely miniaturized EPRoC spectrometers in combination with a permanent magnet (*74*). The injection-locked EPRoC array is embedded in a phase-locked loop (PLL), with a radio-frequency generator (420 MHz) serving as reference, which allows precise control of both MW phase and frequency. The single FM signal is then provided globally by *V*_tune,_ for all VCOs. A 32× multiplier/divider is placed on the chip such that the on-chip MW frequency is 32 × 420 MHz = 13.44 GHz.

## RESULTS

### Simulation of *B*_1_ and *E*_1_

**Fig. 2.**
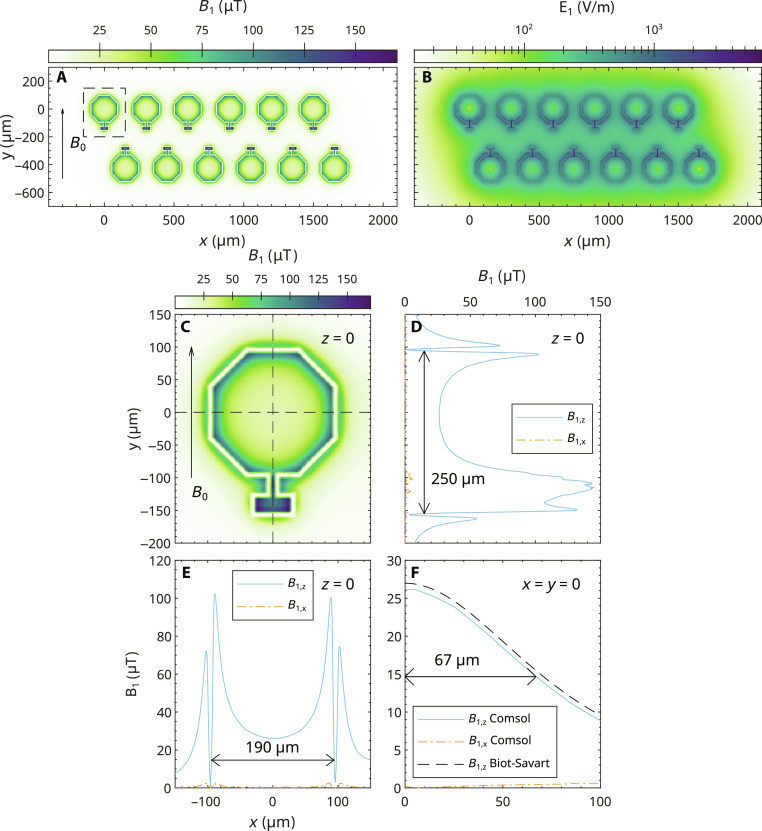
Simulated distribution of the magnetic and electric field of the MW of the 12-coil EPR-on-a-chip array at its surface (*z* = 0). The simulation was performed for a bias current of 5 mA. (**A**) Magnitude of the magnetic field $B_{1}=B_{1,x}{\hat{e}}_{x}+B_{1,z}{\hat{e}}_{z}$ (⊥ to **B**_**0**_). As indicated, **B**_**0**_ is parallel to *y*. The dashed rectangle shows the close-up used in the lower part of the figure. (**B**) Magnitude of electric $E_{1}=E_{1,x}{\hat{e}}_{x}+E_{1,y}{\hat{e}}_{y}+E_{1,z}{\hat{e}}_{z}$ on a log-scale. (**C**) **B**_**1**_ in an enlarged view of a single coil. (**D**) **B**_**1,*z***_ and **B**_**1,*x***_ as a function of *y*. (**E**) **B**_**1,*z***_ and **B**_**1,*x***_ as a function of *x*. (**F**) **B**_**1,*z***_ and **B**_**1,*x***_ as a function of *z* in the center of the coil (*x* = *y* = 0). In addition, **B**_**1,*z***_ calculated with Biot-Savart’s law is indicated. **B**_**1,*x***_ *=* **B**_**1,*y***_ = 0 for Biot-Savart’s law due to rotational symmetry. The double arrows indicate the distance at which the **B**_**1**_ decayed to 10% of the maximum value [~150 μT at *y* ≈ −100 μm in (D)].

The numerically simulated distribution of the magnetic component of the MW ( $B_{1}=\left\|B_{1,x}{\hat{e}}_{x}+B_{1,z}{\hat{e}}_{z}\right\|$ ) of the 12-coil EPRoC array with the minimum available bias current of the EPRoC of 5 mA perpendicular to *B*_0_ ∥ *y* is displayed in Fig. 2A. Please note that the *y* component, $B_{1,y}=B_{1,y}{\hat{e}}_{y}$ , is not shown here as it is parallel to *B*_0_ and would not produce an EPR signal for a Kramers’ system with half-integer spin. The analysis of non-Kramers’ systems, however, may benefit from the **B**_**1,*y***_ component. To reduce computational time, the *B*_1_ field of only one half-coil with the layer structure shown in Fig. 1C on an infinite Si substrate was simulated at 14 GHz (see section Oscillatory magnetic field simulations for more information). To obtain the *B*_1_ distribution of the complete array, the simulated data of this half coil were mirrored, translated, and superimposed. In this way, the mutual inductance between the coils is not considered, which may lead to erroneous values of the *B*_1_ distribution especially between the coils. For instance, assuming two planar coils in a single plane with current flowing in the same direction such that *B*_1_ is generated in the same direction in both coils. Because of symmetry reasons, the *B*_1_ field should be zero at the midpoint of the distance between the centers of two adjacent coils. However, the *B*_1_ distribution inside each coil is only slightly influenced by the neighboring coils since the remaining *B*_1_ of the nearest neighbor (along *x*) is less than 0.5% of that in the center of the coil and only about 10% at the location of the coil trace. *B*_1_ is mainly concentrated inside the conductor loops as seen from Fig. 2A. A close-up of the distribution for one coil is shown in Fig. 2C with cross sections in *x* (Fig. 2E), *y* (Fig. 2D), and *z* directions (Fig. 2F). As seen in the *x* and *y* cross sections, the *B*_1_ is maximum at the inner edges of the trace of the coil with values of ~100 μT and decreases toward the center of the coil at a rate proportional to 1 / *r*, where *r* is the distance to the coil trace. The maximum *B*_1_ lies in the region at about *x* = 0, *y* = −150 μm, where the coil is connected to the varactor (rectangular shape in Fig. 1F) with a magnitude of about 160 μT. Outside the coils, *B*_1_ is minimal, as expected. *B*_1_ in the *z* direction in the center of the coil is much larger than the *B*_1_ in *x* direction as seen in Fig. 2 (D and E). The cross section in *z* direction at *x* = *y* = 0 reaches a maximum at *z* = 0 of about 26 μT. Like in *x* and *y* directions, the contribution of **B**_**1,*x***_ to the total *B*_1_ is negligible. In general, however, **B**_**1,*x***_ is a relevant contribution to the total *B*_1_ and needs to be considered especially at *z* ≫ 0 (see fig. S2 for the distribution at *z* = 20 μm).

The simulation was compared to an analytical solution of *B*_1,*z*_ obtained from the Biot-Savart law applied to a circular current loop. The magnetic field on the center axis [*B*_1,*z*_(*x* = 0, *y* = 0, *z*)] of a circular current loop with a radius, *R*, equal to that of one coil of the EPRoC (*R* = 100 μm) with the current in the coil, *I*_coil_, can be calculated as

$$B_{1,z,\text{Biot}-\text{Savart}}\left(z\right)=\frac{1}{2}\frac{\mu_{0}I_{\text{coil}}}{2}\frac{R^{2}}{{\left(R^{2}+z^{2}\right)}^{3/2}}$$ (4)

where μ_0_ is the vacuum permeability. The factor 1/2 takes into consideration that only half the *B*_1_ magnitude is resonantly interacting with the spin ensemble due to the two counter-rotating MW fields in the rotating frame. The numerical expression of Eq. 4 yields the same line shape as the numerical simulation (COMSOL), shifted by less than 1 μT, as seen in Fig. 2F.

The *B*_1_ distributions shown in Fig. 2 (D to F) indicate that *B*_1_ decays to approximately 10% of its maximum value of ~150 μT (located at *x* = *z* = 0 and *y* ≈ −100 μm) outside a volume of 190 μm by 250 μm by 67 μm (3.2 nl) around the center of the coil. Under nonsaturating conditions, this drop would correspond to a signal amplitude reduction by 99% as seen in Eq. 3. In this case, the square of the unitary magnetic field, $B_{1u}^{2}$ , in the numerator dominates the signal of Eq. 3, while the so-called saturation term in the denominator is negligible, i.e.,  $\gamma^{2}B_{1}{\;}^{2}T_{1}T_{2}\ll 1$  (no saturation).

Figure 2B shows the electric component of the MW of the 12-coil array for a bias current of 5 mA simulated as described before. The simulation reveals a concentration of the *E*_1_ at the small gap between the conductors with values up to 3 kV/m. Inside and outside the conductor loops, the values are about one order of magnitude lower.

For a TE-102 cavity resonator with a *B*_1_ available for EPR of ~27 μT in its center (corresponds to ~40-mW MW input power to a Bruker ST4102 cavity), a maximum *E*_1_ of ~21 kV/m about 10.5 mm away from the center of the cavity is expected (*75*, *76*), which is about one order of magnitude larger than the *E*_1_ observable with the EPRoC sensor for a similar *B*_1_ in the center (see Calculation in the Supplementary Materials). This implies the use of sample tubes with a diameter ≪  10.5 mm for resonator-based EPR, such as 4-mm outer diameter (3-mm inner diameter) tubes to avoid excessive MW absorption caused by *E*_1_. Even with these tubes about 25% of the max., *E*_1_ is remaining at the tube wall.

As the *E*_1_ values of the EPRoC are of the order of 10^−1^ kV/m inside the conductor loop, samples smaller than the coil diameter are expected to experience lower nonresonant MW absorption through the *E*_1_ field than larger samples. In addition, extended lossy samples such as polar liquid samples and generally samples with a large dielectric constant may profit from the overall lower *E*_1_ of the EPRoC compared to resonator-based EPR.

### EPRoC spectra of BDPA and a-Si

**Fig. 3.**
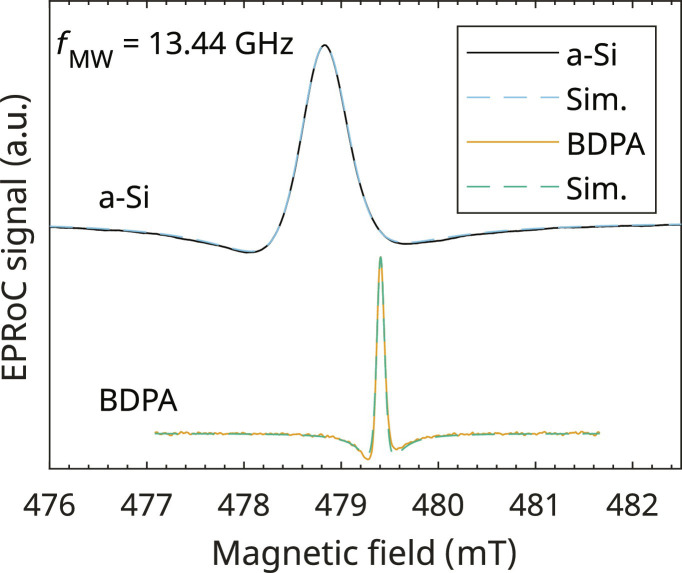
Field-swept EPRoC FM spectra of a-Si and BDPA with respective simulations. (**Top**) a-Si; (**Bottom**) BDPA. The spectra were acquired with a bias current of 5 mA. For PSD, the MW frequency was modulated with a frequency of 75 and 100 kHz for a-Si and BDPA, respectively, and peak-to-peak modulation amplitude of 5.3 MHz (~0.19 mT) and 0.96 MHz (~0.03 mT) for a-Si and BDPA, respectively.

Figure 3 shows the experimental EPRoC FM spectra (solid lines) of the thin-film a-Si sample and a BDPA sample with respective spectral simulations. As expected for the FM signal of the EPRoC, both spectra are the first derivatives of a dispersion signal due to the phase-sensitive detection (PSD) of the real part of the complex sample susceptibility of a single line. Both spectra show a slight asymmetry, most likely due to the oscillator-based detection and its bidirectional coupling of the spin system and MW as it is seen in all EPRoC FM spectra published previously (*54*, *55*, *57*, *59*, *60*, *65*, *66*). The spectra were simulated (see dashed lines in Fig. 3) using the EasySpin software package (*77*) assuming a spin-1/2 system with isotropic *g*-value obtained from literature (*78*, *79*) with convolutional Gaussian and Lorentzian broadening (i.e. pseudo-Voigtian), with the peak-to-peak linewidths, Δ*B*_pp,G_ for Gaussian and Δ*B*_pp,L_ for Lorentzian broadening, respectively, being least-square fitted to the experimental data. Please note that the linewidths presented here correspond to the peak-to-peak linewidths of a first-derivative EPR absorption spectrum and was used to stick to the usual convention used in the EPR community; therefore, the linewidths cannot be directly read off the spectra displayed here. The parameters used for the simulation can be found in Table 1.

**Table 1.** Parameters of the spectral simulations of BDPA and a-Si. Only the linewidth parameters were least-square fitted, while the other parameters were kept constant. The literature *g* value were taken from (*79*) and (*78*) for BDPA and a-Si, respectively.

Quantity	BDPA	a-Si
*S*	1/2	1/2
*g*	2.003	2.0055
Δ*B*_pp,G_ (mT)	0.00	0.24
Δ*B*_pp,L_ (mT)	0.10	0.49

The spectrum of the BDPA shows a Lorentzian behavior only with a peak-to-peak linewidth of 0.1 mT. In (*80*), an intra-grain distribution of *T*_2_ was found for BDPA grains from one batch to be in the range of 80 to 160 ns in X-band. Assuming a relaxation-determined broadening of the BDPA, the linewidth may be determined by

$$\Delta B_{\text{pp},L}=\frac{2}{\sqrt{3}\gamma T_{2}}$$ (5)

where γ is the electron gyromagnetic ratio. Using Eq. 5, the linewidths in X-band are in a range from ~0.04 to ~0.08 mT, which is about 60 to 20% smaller compared to the presented data. Possible reasons for the broadening include saturation as well as frequency-dependent broadening mechanisms, e.g., disorder-induced *g* strain. As spectra obtained for BDPA at Q-band do not show additional broadening, the former is unlikely. Saturation, however, may play a role as the *B*_1_ magnitudes seen in Fig. 2A are up to ~150 μT close to the conductors of the coil and ~27 μT in the center of the coil. Using *T*_1_ = 110 ns from (*81*) and *T*_2_ in the range as stated above, the sample is saturated even in the center of the conductor loop, with possible saturation broadening.

The a-Si is best fitted with a (pseudo) Voigtian lineshape with a “total” linewidth of 0.6 mT, with Gaussian and Lorentzian contributions of 0.10 and 0.49 mT, respectively, which is in good agreement with literature values of similar samples (*82*). Here, the total linewidth ranged for undoped unhydrogenated a-Si between 0.4 and 0.8 mT in X-band depending on the spin density and sample preparation. EPR spectra obtained at S-, X-, and Q-band revealed that both Gaussian and Lorentzian contributions increase linearly with MW frequency. While the Lorentzian contribution of the linewidth obtained with the EPRoC resulted in the expected value from the linear increase observed with respect to frequency, the Gaussian contribution was much larger than expected (62%). This additional broadening may originate from saturation effects, which are particularly pronounced in the EPRoC for samples that protrude toward the traces of the VCO coils where *B*_1_ fields are greatest.

Saturation has been observed to be routinely present when performing CW EPRoC measurements even for samples with relatively fast relaxation rates such as BDPA or a-Si. The effect of saturation may be alleviated either by decreasing the magnitude of *B*_1_ or by decreasing the time spent on resonance. The former may be achieved by, e.g., using the high-electron mobility transistor (HEMT) technology instead of complementary metal-oxide semiconductor (CMOS) technology, which has been discussed in (*59*). This allowed for approximately 30× lower *B*_1_ compared to the CMOS EPRoC used in this work, would however, require a complete redesign of the EPRoC. The latter approach of reducing the time spent on resonance is used in rapid-scan EPR (RS-EPR) (*83*). Quantitative RS-EPR on hydrogenated a-Si exhibited an improved sensitivity compared to CW-EPR by more than one order of magnitude (*4*). Rapid frequency scan EPR experiments using AM detection recorded with the same EPRoC as in the herein reported experiments showed the possibility of recording unsaturated AM spectra of BDPA obtained by Fourier deconvolution of RS-EPRoC transients (*61*). Since this work focused on the FM signal, which profits from the array concept, the possibly saturated CW-EPRoC FM spectra of BDPA and a-Si were used instead. For the experiments discussed in the remainder of the manuscript, the peak-to-peak amplitudes of field-swept EPRoC spectra of BDPA and a-Si shown in Fig. 3 were extracted and plotted.

### EPR signal distribution in a single coil

**Fig. 4.**
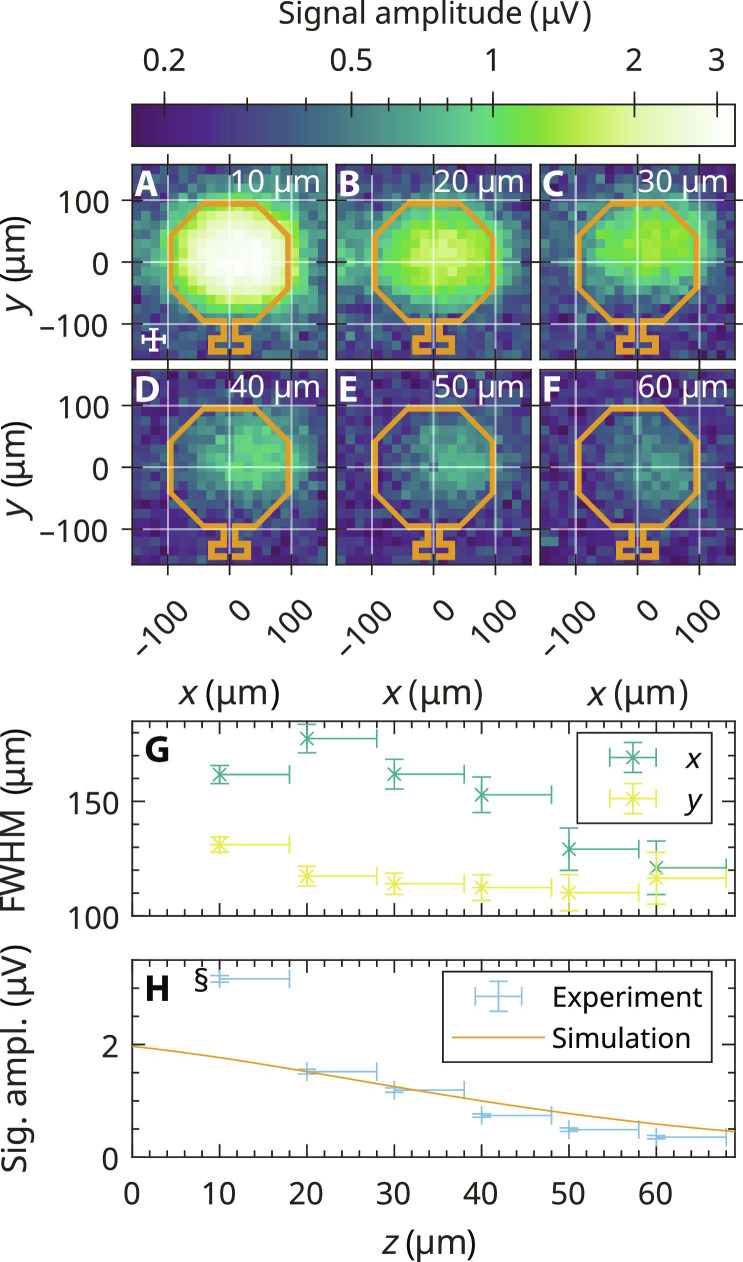
Mapping of EPR signal in one of the outermost coils of the EPRoC array using a single grain of BDPA. (**A** to **F**) Peak-to-peak signal amplitude at a distance *z* from the sample to the surface of the chip. The cross in (A) indicates the approximate sample size of 35 μm in each direction, which contains approximately 10^14^ spins. The octagons illustrate the location of the conductor and are shown to scale with a diameter of 200 μm and a thickness of 8.5 μm. To ensure a proper visibility of the data, a log-scale was used for the color map. (**G**) FWHM in *x* and *y* directions as obtained from a 2D Gaussian fit of the maps shown in (A) to (F). (**H**) Signal amplitude as a function of the distance of the sample to the surface of the EPRoC. Please note the comment about the absolute values of the *z* axis in the text. §, outlier as described in the text.

First, the dependence of the FM signal amplitude on the sample position in a single coil of the EPRoC array was investigated. The mapping of the EPR signal of one of the outermost coils of the EPRoC array, recorded at different heights above the surface of the coil from ~10 to ~60 μm using a single grain of BDPA (⌀ ≈ 35 μm, ~10^14^ spins) is shown Fig. 4 (A to F), displaying the peak-to-peak amplitude of the FM signal. For each data point shown in the plots, a first-derivative FM spectrum was recorded from which the peak-to-peak amplitude was extracted. To compare the sizes of the sample and the coil, a cross is displayed on the bottom left, representing the sample size, while the shape of the VCO coil with a diameter of 200 μm is also indicated.

The experimental data demonstrate that the EPRoC array is primarily sensitive inside the conductor loop, while the signal amplitude outside the coil is vanishingly small. The maps were least square fitted with a 2D Gaussian profile to obtain the full width at half maximum (FWHM) of the peak, the result of which is displayed in Fig. 4G. The values of the FWHM exhibit an asymmetry in *x* and *y* directions with values of about 160 μm in the *x* direction and 120 to 130 μm in the *y* direction. In addition, the FWHM in the *x* direction decreases with increasing *z* to about 120 μm FWHM at *z* = 60 μm, while the FWHM in *y* direction does not change considerably.

From the data presented, the sensitive volume of one of the coils of the 12-coil EPRoC array for BDPA may be estimated. On the basis of the shape of the observed BDPA signal amplitudes, we assume an ellipsoidal cylinder with a semi-major axis, *a*, of 80 μm, semi-minor axis, *b*, of 62.5 μm, and a height, *h*, of about 60 μm

$$V_{\text{sens},\text{coil}}=\pi \times a\times b\times h\approx 0.9\;\text{nl}$$ (6)

The experimentally determined sensitive volume is about a factor of 3 lower than the “sensitive” volume calculated from the simulated *B*_1_ distribution in section Simulation of *B*_1_ and *E*_1_. A possible explanation for the discrepancy is the saturation behavior of the BDPA sample, which decreases the signal amplitude as discussed in section EPRoC spectra of BDPA and a-Si since the BDPA grain saturates predominately close to the trace of the coil.

In Fig. 4H, the height dependence of the signal amplitude is shown, which was extracted from the mapping in (Fig. 4, A to F). At *z* > 60 μm, the FM spectra decreased in amplitude below the levels of the noise and the baseline. The observed signal amplitude decreases nonlinearly with increasing distance from the chip surface due to the decreasing *Β*_1_, which is confirmed by a simulation of the signal amplitude using Eqs. 2 and 3, which consider the simulated *Β*_1_ distribution, the literature values for the relaxation times of BDPA, and the sample size (35 μm).

The signal amplitude of the first data point at *z* = 10 μm (denoted with § in Fig. 4) is about a factor 2 larger than expected from extrapolating the values of the remaining data points. Even when considering the error of the *z* axis, it does not fit to the rest of the dataset. Therefore, the first experimental data point in Fig. 4H at *z* = 10 μm was omitted, and the simulation was instead normalized to the signal amplitude recorded at *z* = 20 μm. The simulated signal amplitudes are in good agreement with the recorded spectra even after consideration of the uncertainty in the absolute position in the *z* axis apart from the first data point. The decrease in signal intensity in the recorded spectra is quite linear for *z* ≥ 20 μm. The former explanation, however, cannot explain the much higher signal amplitude of the first data point at *z* = 10 μm and it seems that the theory presented in Eq. 3 is incomplete.

### EPR signal distribution of three coils

**Fig. 5.**
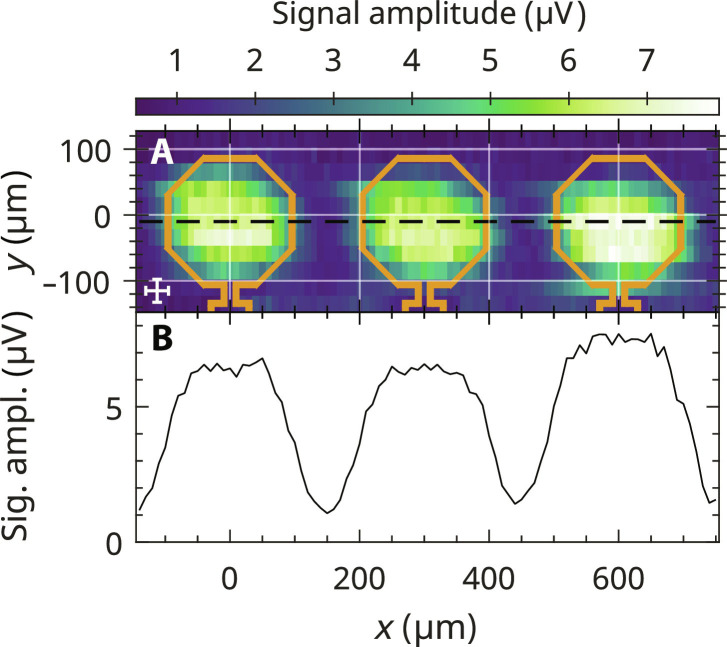
Mapping of three coils of the EPR-on-a-chip VCO array at *z* ≈ 20 μm above the EPRoC. (**A**) Map of the normalized peak-to-peak FM amplitude of three coils of the 12-coil EPRoC array. The cross (bottom left) indicates the approximate sample size of 35 μm in each direction containing approximately 10^14^ spins. The octagons illustrate the conductors each with a diameter of 200 μm and a thickness of 8.5 μm. (**B**) Cross section of the map taken in the center of the three coils indicated by the black dashed line in (A). All three coils show similar signal amplitude.

To investigate the behavior of the FM signal over multiple coils, mapping of three neighboring coils of the 12-coil EPRoC array was performed with a grain of BDPA (⌀ ≈ 35 μm, ~10^14^ spins) at a distance of about *z* = 20 μm to the surface of the EPRoC. The peak-to-peak amplitude of the FM recorded signal with respect to sample position in the *x*-*y* plane is shown in Fig. 5A. A cross is displayed on the bottom left, representing the sample size, while three VCO coils with a diameter of 200 μm are indicated by octagons with widths (8.5 μm) shown to scale to represent the thickness of the conductors in the EPRoC device. The experimental data show that the EPRoC array is primarily sensitive inside the conductor loops as was observed in section EPR signal distribution in single coil and is independent of sample placement given that the FM signal is recorded in all coils. Outside the coils, the EPR signal is vanishingly small. Figure 5B shows a cross section through the center of the three coils, taken from Fig. 5A. Inside the coils, the FM signal amplitude plateaus with a width of about 120 μm and an amplitude value of ~7 μV with a coefficient of variation in this region of less than 3%. The minute differences in signal amplitudes and FWHM measurements in the differing plateau regions originate from slight misalignments in sample placement. In between the coils, the signal amplitude decreases to about 1 μV. The plateaus were fit with a 2D Gaussian function to determine the FWHM of each plateau in the *x* and *y* directions, which is asymmetric as was observed previously with larger values for both FWHM in *x* and *y* directions of ~180 and ~140 μm, respectively. One possible reason for the larger FWHM is the increase in signal amplitude by a factor of ~3.5 compared to the mapping of one coil. This discrepancy may be further explained by large errors (~80%) in the estimation of the sample volumes. Because of the small dimensions of the BDPA samples and the uncertainties arising from sample handling, estimation of the shape and volume of the sample is generally challenging; however, the samples were selected because of their notably smaller sample volume relative to the diameter of the EPRoC coils such that the criteria for a point-like sample were adequately achieved. The procedure performed in this work is described in section Simulation of EPR signal dependence on partial utilization of the EPRoC array.

From the data presented in Figs. 4 and 5, the sensitive area of the entire EPRoC VCO array may be determined, such that the coils of the VCO array may be regarded as independent detectors with respect to the *B*_1_ field. For the complete array with 12 coils (*n*_coil_), the sensitive volume is therefore estimated to be 11.3 nl.

From the experimental data of the one- and three-coil mapping shown in Figs. 4 and 5, a decrease of the signal amplitude was found when moving the sample from the center to the trace of the coil. In (*48*), a similar signal reduction was observed when moving the BDPA sample away from the central position of the resonator (see Fig. 3D) similar to the behavior observed here. Their results, however, are only partially comparable to the herein reported work, since the investigated BDPA sample was much larger than the sensitive region of the inverse anapole resonator (15 μm by 20 μm by 5 μm). Therefore, in (*48*), the sample when placed in the center of the resonator filled the complete sensitive volume, while the sample when placed offset to the center only partially filled the sensitive volume, effectively decreasing the filling factor, η, as defined in Eq. 1. In addition, the *B*_1_ in the position of the coil where the sample was present was lower than in the center, which was confirmed by a saturation measurement at both positions, additionally decreasing the filling factor. The different *B*_1_ values in the center and the offset of the center are caused by the inverse anapole design used in their work.

**Fig. 6.**
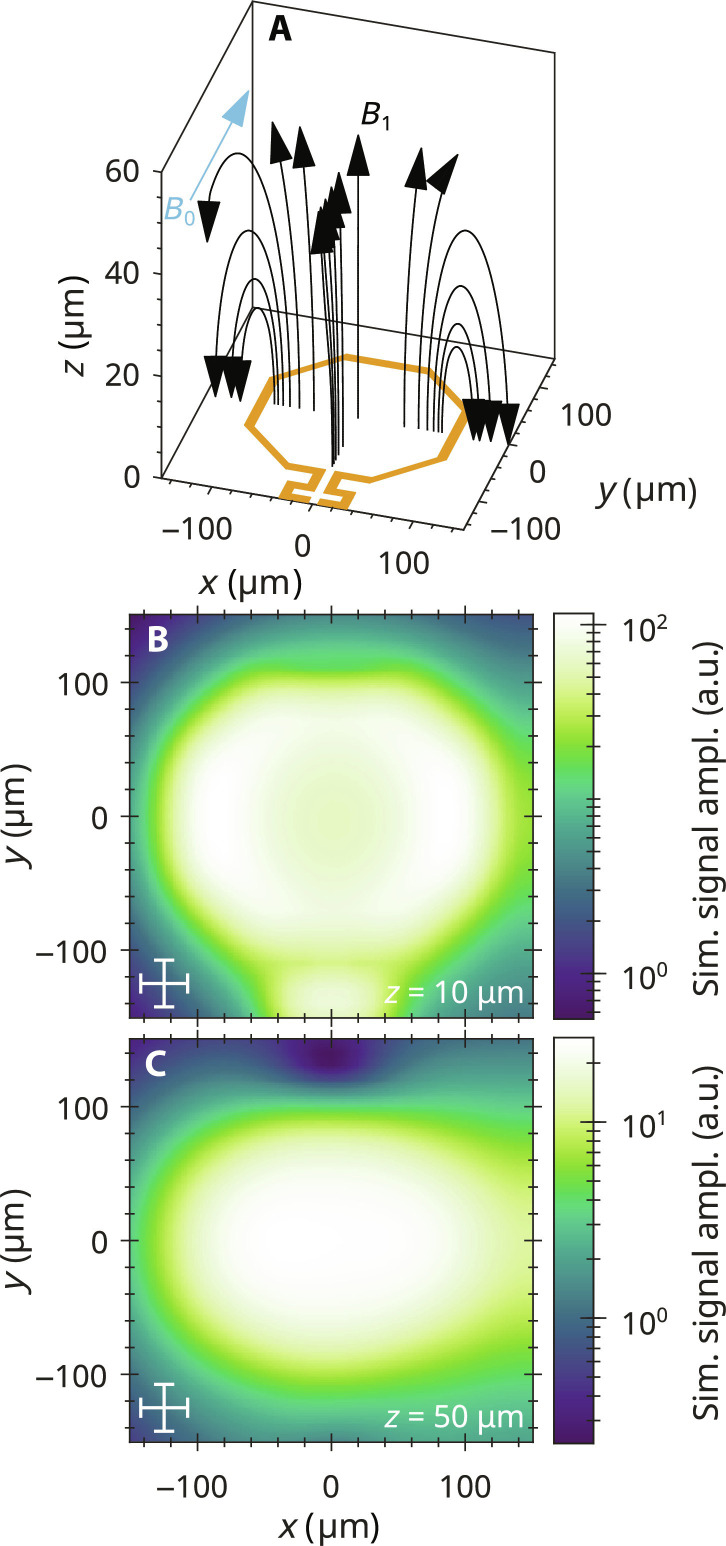
Effect of the inhomogeneous *B_1_* on the signal amplitude of a point-like BDPA sample. (**A**) Magnetic field lines (black) indicating the **B**_**1**_ inhomogeneity of the coil of a single EPRoC VCO leading to an asymmetric shape of the map of the signal amplitude of an extended sample. (**B** and **C**) Simulated map of the FM signal amplitude of a cubic-shaped sample of BDPA with an edge length of 35 μm (indicated by the cross in lower left corner) and relaxation times of *T_1_* = 110 ns and *T_2_* = 100 ns at distances of 10 μm and 50 μm to the surface of the EPRoC taking the **B**_**1,*x***_ component of the MW also into account. Note that the color scale is logarithmic for a better comparison with the experimental data shown in Fig. 4.

An asymmetry of the extent of signal amplitude in *x* and *y* directions is observed in the experimental data of the one-coil and three-coil mapping shown in Figs. 4 and 5, respectively. This asymmetry may be explained by the inhomogeneous *B*_1_ distribution of the EPRoC in each coil with relevant contributions from all MW fields perpendicular to *B*_0_ as shown in Fig. 6A. A simulation of the mapping of the signal amplitude of a cubical BDPA sample with an edge length of 35 μm and relaxation times of *T*_1_ = 110 ns and *T*_2_ = 100 ns at a distance of 10 μm in Fig. 6B shows a similar asymmetry indicating that *B*_1,*x*_ is a relevant contribution to the total *B*_1_ in addition to *B*_1,*z*_, both perpendicular to *B*_0_, which needs to be considered even for point-like samples. In addition, the simulation shows an increase of the signal amplitude when moving the sample from the center toward the conductor path of the coil, which is especially pronounced in the *x* direction (factor of ~2) due to the contribution of *B*_1,*x*_ to the total *B*_1_ and an increase at about (0 μm, −125 μm) originating from the relatively large *B*_1_ in this area. This behavior is not seen in the experimental data presented here but in the experiments in (*59*), where an increase of the signal amplitude by a factor of approximately 100 was found when moving a small BDPA sample from the center of the coil toward the outer edge. This increase was attributed to an increased filling factor, η, defined in Eq. 1. In this equation, the denominator is constant as the volume is integrated over all space while the numerator scales with *B*_1_^2^ as the *B*_1_ inside the sample changes when it is moved. This in turn linearly affects the signal amplitude if the sample is unsaturated. The factor of ~100 fits well with the *B*_1_ magnitudes in their experiments of 0.4 μT in the center and 4 μT close to the trace of the coil (factor of 10 in *B*_1_). These *B*_1_ values are much lower (factor of 35 to 65) compared to the EPRoC used in this work due to a larger coil size and the utilization of an HEMT in their Colpitts oscillator circuit, allowing for lower minimum coil currents. In the experiments reported in this report, the *B*_1_ magnitudes are approximately 27 μT in the center and 100 μT close to the trace at *y* = 0 (see Fig. 2E). Hence, an increase of the signal amplitude of ~10 is expected from the filling factor, while the simulation in Fig. 6B only shows an increase by factor of ~2. This, however, may be attributed to saturation of the BDPA sample due to the larger *B*_1_ magnitudes of the used EPRoC. As mentioned above, the experimental data do not show an increase of the signal amplitude when moving the sample toward the trace of the coil contrary to the simulation at *z* = 10 μm, where a maximum of the signal amplitude is found at two positions at *y* = 0 and *x* ≈ ±90. Further simulations at larger distances revealed that these two maxima of the signal amplitude plateau at approximately 50 μm as shown in Fig. 6C. This indicates that the sample in the experiment may be approximately 40 μm further away from the surface of the EPRoC as initially assumed, i.e., 50 μm instead of 10 μm for the first data point. Consequently, the sensitive height for BDPA would then be approximately 40 μm larger than initially assumed and is therefore 100 μm. The sensitive volume for BDPA would then be approximately 1.6 nl for one coil and 19.2 nl for the complete array.

In general, the effective sensitive volume of the EPRoC depends on the relaxation times of the investigated sample due to saturation of the EPR signal at different positions in space as seen in Eq. 3. In this equation, the EPR signal is calculated as a volume integral over all electron spins in the sample with varying *B*_1_ magnitudes depending on their position. Two contributions of *B*_1_ compete at each point. While the *B*_1u_ in the numerator increases the signal amplitude independently of the relaxation times, the contribution of the saturation factor in the denominator, s = γ^2^*B*_1_^2^*T*_1_*T*_2_, depends on the relaxation times. For samples with (very) short relaxation times, the sensitive volume is therefore larger than for samples with long relaxation times as the spectra saturate more easily. In this case, the lines in the EPR spectra may be broadened such that the signal amplitude does not increase linearly with *B*_1_.

In general, one way to reduce the inherent *B*_1_ inhomogeneity of planar microresonators perpendicular to the surface was presented in (*47*), where a 3D self-resonant helix with good *B*_1_ homogeneity along the axis of the helix was used as an EPR resonator. A similar improved *B*_1_ homogeneity for the EPRoC may be achieved by stacking multiple EPRoC (array) sensors with holes within each coil so that thin sample capillaries may be inserted. This concept was introduced in (*84*), in which a single-coil EPRoC with a hole drilled within the coil was presented.

### Height dependence of the signal amplitude of an a-Si sample

Having determined the spatial properties of the EPRoC FM signal with a point-like sample for which only one coil of the EPRoC VCO array was used at a time, it was then necessary to investigate the behavior of the EPRoC array FM signal when all coils are used at the same time. This was performed by recording the FM signal of a thin film a-Si sample, with lateral extension being much larger than the whole array and, hence, covers all VCO coils of the array simultaneously. Here, the height dependence of the EPRoC FM signal could be investigated rather precisely because the translational movement of the a-Si thin film in the *x* and *y* directions does not change the number of spins present in any single coil due to the homogeneity of the thin film and the size of the film relative to the coil array.

**Fig. 7.**
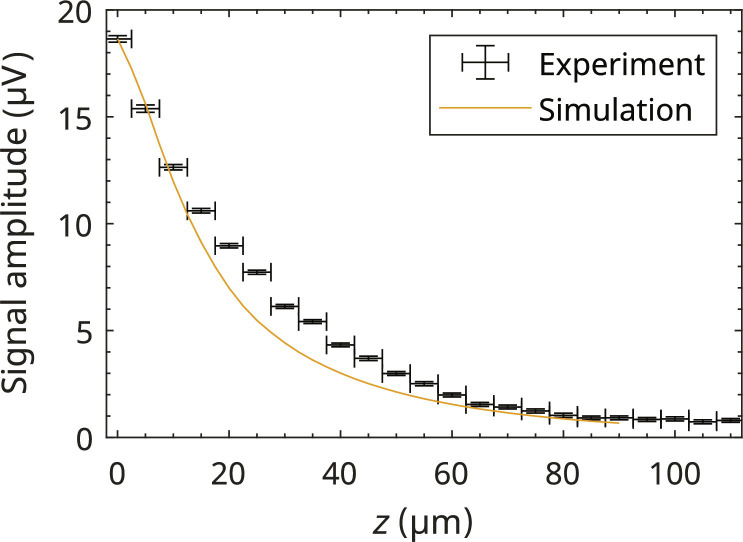
Height dependence of the EPRoC FM peak-to-peak amplitude of an a-Si thin film (15 μm) sample. In addition, a simulation of the signal amplitude of the a-Si sample is shown. For the simulation, a cuboidal a-Si sample with edge lengths of 2 mm by 1 mm by 0.015 mm and relaxation times of *T*_1_ = 200 ns and *T*_2_ = 20 ns was assumed. Please note that the simulation is normalized such that the value at 0 μm corresponds to the experimental value at this position.

The sensitive height of the EPRoC array was determined by recording multiple EPR spectra at varying distances of the sample relative to the chip. The peak-to-peak signal amplitude, calculated as the amplitude difference between the maximum and the minimum of the line in the recorded spectrum, and noise, calculated as the SD of the baseline region, are plotted in Fig. 7 as a function of the distance from the EPRoC. At *z* = 0, the a-Si sample was in direct contact with the EPRoC VCO array, which was confirmed using a digital single-lens mirrorless (DSLM) camera placed approximately 50 cm away from the EPRoC and outside of the field of the magnet. Above about 100 μm, the EPR signal is indistinguishable from the baseline noise, yielding a sensitive height of about 100 μm, which is in good agreement with the value determined from the mapping experiments with BDPA.

To compare the experimental data to theoretical considerations, the expected FM signal amplitude was simulated using the *B*_1_ distribution discussed in section Simulation of *B*_1_ and *E*_1_, which is also shown in Fig. 7 for the a-Si sample. For the simulation, a cuboid (2 by 1 by 0.015 mm^3^) of a-Si with relaxation times *T*_1_ = 200 ns and *T*_2_ = 20 ns was assumed (see the simulation of the one-coil map). The sample was uniformly distributed in voxels (2.5 by 2.5 by 2.5 μm^3^), and the first-derivative dispersion EPR spectrum was calculated using Eq. 2. For each sample position in the *z* direction, the spectra were summed up, and the peak-to-peak amplitude was obtained. The simulated signal amplitude was normalized to coincide with the first data point at *x* = 0 in Fig. 7.

The simulation of the dependence of the signal amplitude on the distance to the surface of the EPRoC using all coils with an a-Si sample shows a good agreement with experimental data. The slight discrepancies between ~20 and 60 μm, where the simulated signal amplitude is lower than the experimental data, could be explained by a slightly tilted sample whose surface is not exactly parallel to the surface of the EPRoC. In this case, one edge would be closer to the sensor area than expected and hence would produce a larger signal amplitude. In general, the absolute *z* values should be seen as a “rough” estimate as the determination of the absolute distance between the EPRoC and the sample can only be performed via high-resolution photographs of the sample in comparison to the setup and indirectly from the motor positions.

### EPR signal dependence on partial utilization of the EPRoC array

**Fig. 8.**
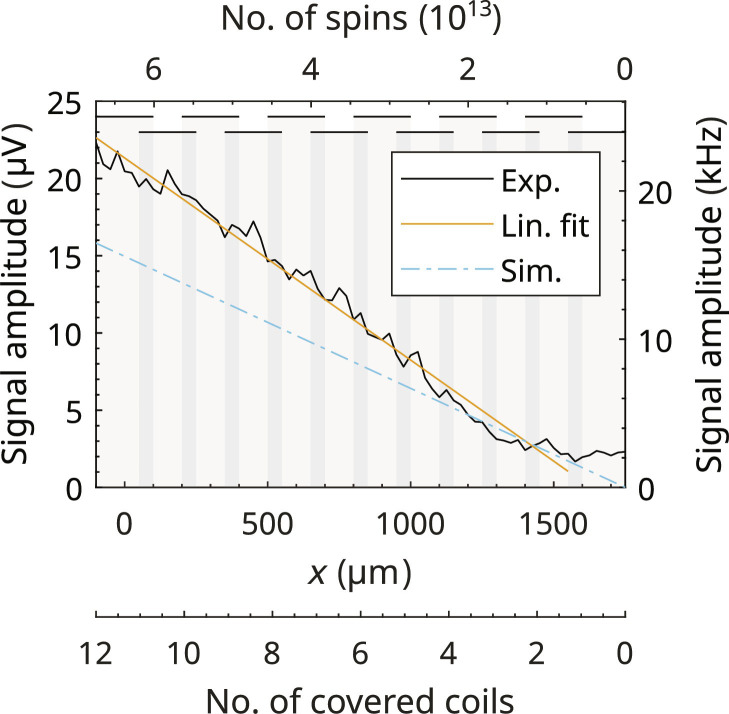
Dependence of the signal amplitude of the FM spectrum on the number of coils occupied. To change number of occupied coils (bottom *x* axis), the a-Si was moved sample along the *x* direction (middle *x* axis). The top *x* axis displays the approximate number of spins in the sensitive area of the sensor, assuming that no signal is detected outside of the coils. The horizontal lines at the top represent the coils on the chip (compared to the layout in Fig. 1D). The number of coils covered by the sample decreases from the left (*12*) to the right (0), where no coils are covered by the sample.

To determine experimentally the effects of injection-locking of the EPRoC array upon partial filling of the VCOs, the dependence of the signal amplitude was recorded when the Si sample was moved horizontally across the EPRoC array sensors along the *x* axis at *z* = 0 as shown in Fig. 8. This procedure allowed the relative filling of the sensitive area of the EPRoC array to be varied, while the peak-to-peak amplitude was determined from the recorded derivative spectrum. The sample was positioned such that the center of the sample in the *y* direction was at *y* = 0. Through the movement of the sample, a decreasing fraction of the sensitive area of the EPRoC array was covered by the a-Si thin film sample while recording the EPR spectrum at each *x* position. At *x* = −100 μm, the sample covered all 12 coils, while at *x* = 1750 μm, no coils were involved.

From the number of coils covered, the approximate total number of spins inside the sensitive area (Fig. 8, top *x* axis) was calculated using the sensitive area of the EPRoC determined from previous measurements, as described above, and from the spin density of the a-Si sample considering a thickness of 15 μm (see section Oscillatory magnetic field simulations for more information). As the sensitive volume depends on the relaxation times of the investigated sample, an error may be introduced by using the sensitive volume determined for BDPA for the a-Si sample. The relaxation times of both samples, however, have the same order of magnitude, so that the possibly introduced error seems acceptable and we can assume similar sensitive volume for both samples. For simplicity, the sensitive volume for the a-Si was assumed to be cylindrical with a height of the a-Si thin film (15 μm) and an elliptical ground plane with semi-minor and semi-major axes as determined before. For each data point shown in Fig. 8, an EPR spectrum was recorded from which the peak-to-peak derivative signal amplitude at the VCO-array’s tuning voltage (*V*_tune_) was determined (Fig. 8, left *y* axis). If only two coils or less (*x* ≥ 1500 μm) are covered by the a-Si thin film sample, the signal amplitude is indistinguishable from the baseline noise of the spectrum. With more than two coils covered (*x* ≤ 1500 μm), a linear increase in the signal amplitude is observed. As explained in section EPR-on-a-chip setup, since the used modulation frequency of 75 kHz is well inside the 10-MHz bandwidth of the PLL used to control the VCO array’s phase, any spin-induced frequency shift of the VCO array is faithfully converted to a shift in its tuning voltage by a conversion factor of about 1.04 GHz V^−1^ (also called the VCO gain). The EPR-induced frequency signal of the VCO array (Fig. 8, right *y* axis) was hence calculated from the *V*_tune_ signal amplitude and the said VCO gain. When all coils are covered by the sample, the peak-to-peak frequency signal of the VCO due to the EPR is about 22.3 kHz. The slope of the linear fit may be interpreted as the frequency signal of the VCO per spin, which was found to be approximately 0.33 pHz spin^−1^ when ~6.7 × 10^13^ spins are present in all VCO coils of the array.

The peak-to-peak frequency signal as a function of the number of covered coils of the injection-locked array was simulated with the harmonic balance-based periodic steady state analysis of the circuit simulator SpectreRF (Cadence Design Systems) using the same simulation procedure as described in (*60*) for an 8-coil array EPRoC, the result of which is plotted in Fig. 8 for a 12-coil array. For the simulation, the same parameters as for the experiment (bias current, MW frequency) were used and the properties of the a-Si sample (relaxation times, spin density, and sample volume) were considered. Section Simulation of EPR signal dependence on partial utilization of the EPRoC array contains a more detailed explanation of the simulation. Similar to the experimental data, a linear increase of the frequency as a function of the used coils is found with a maximum peak-to-peak frequency signal of approximately 16.5 kHz when all coils are covered, corresponding to 0.24 pHz spin^−1^.

As seen in Fig. 8, the frequency signal amplitude increases linearly with the number of coils filled with sample, which shows the potential to use the EPRoC array for quantitative EPR even in applications where the sample is not homogeneously distributed within the sensitive area of the array if the sample size is known.

To explain the behavior of the relative sample occupancy of the VCO array qualitatively, two contributing factors must be considered: the filling of the single coils of the VCOs and the injection-locking of the VCOs in the EPRoC array. For materials obeying Curie magnetism, the magnetic susceptibility is linearly proportional to the number of paramagnetic centers in the sample. Since the FM signal is proportional to the static magnetic susceptibility, a linear increase of the signal intensity upon covering or filling of a single coil is expected. The effect of the injection-locking of the VCOs was simulated in (*60*), which revealed that in injection-locked VCO arrays previously used for EPR, the signal amplitude of the global FM signal is reduced if not all coils are covered with paramagnetic sample. The total frequency shift for a paramagnetic sample with a number of spins, *n*_spin_, is similar in both a single VCO and in an injection-locked VCO array, for which the total number of spins is distributed over all coils.

The simulation of the frequency shift as function of the number of used coils of the EPRoC array shows a linear increase similar to that of the experimental data. While in the experiment, a convolution of the filling of the coils and the injection-locking is found as explained above, only the latter is considered in the simulation. Using the same experimental settings for the simulation and considering the relaxation times, the sample volume and spin density of the sample, we find that the frequency signal amplitude is approximately 16.5 kHz when all coils are used in the simulation, which is about 35% smaller than that determined experimentally. This difference may be explained by the limitations of the simulation that did not consider the filling of the coils mentioned above and the *B*_1_ inhomogeneity. In addition, the sample volume was determined from the BDPA sample and not an a-Si sample.

## DISCUSSION

In conclusion, we have demonstrated the accessible *B*_1_ distribution of a VCO-based EPRoC array detector enroute to quantitative EPR-on-a-chip applications by mapping the *B*_1_ field distribution in three dimensions using a point-like grain of BDPA and a thin-film sample of a-Si. In these experiments, the sensitive area of a single VCO was found to be approximately cylindrical with an elliptical ground plane with a semi-major axis, *a*, of 80 μm and semi-minor axis, *b*, of 62.5 μm and the sensitive height to be around half the coil diameter (100 μm), showing that the sensitive region is mainly inside the coils with a sensitive volume of ~1.6 nl per coil. The experimental data were compared to simulations using the steady-state solution of Bloch’s equations in combination with finite-element simulations of the *B*_1_ distribution, which were found to be in good agreement. It was also found that the contribution of *B*_1,*x*_ to the total *B*_1_ may not be neglected, which explains the deviations from purely cylindrical distributions observed within the recorded data. Since the finite-element simulations of the *B*_1_ distribution in combination with a simulation of spatial dependence of the EPR signal amplitude are in good agreement with the experimental data, it may be sufficient for future EPRoC designs to estimate the *B*_1_ distribution using only the simulated data.

In addition, the dependence of the FM signal, when only partially covering the sensor, was determined. Here, a linear increase of the signal amplitude was observed indicating that even upon incomplete covering of the EPRoC array with sample, quantitative EPR is still possible if the spatial sample distribution is known. Simulations confirmed the linear increase as a function of the number of used coils. These results similarly support the possibility of quantitative measurements in samples where the distribution of spins within the sample is inhomogeneous.

Although we have used an electromagnet in this study for practical reasons, the ability to use small permanent magnets via frequency-swept CW-EPR as shown in previous publications, coupled with its small size and power consumption, makes for great flexibility in EPRoC applications. In the future, EPRoC sensors may also be integrated into various complex and harsh sample environments such as electrochemical cells and redox flow batteries, enabling in situ and operando EPR measurements that have previously been inaccessible. This includes handheld devices for in-the-field multi-line fingerprinting applications in chemistry, medicine, biology, material science, and physics, in addition to quantitative sensor applications for food quality control, energy storage solutions, and point-of-care medical diagnostics.

## MATERIALS AND METHODS

### EPR-on-a-chip setup

The schematic of the used experimental configuration is thoroughly described in (*61*) and briefly described here. A printed circuit board containing a 12-coil EPRoC array was placed between the magnet poles of the electromagnet of a Bruker ESP300 X-band spectrometer controlled with a home-written LabVIEW software. The accessible sweep range of the EPRoC used in these experiments is approximately 12.0 to 14.4 GHz giving a full width of 2.4 GHz, which is equivalent to 85.6 mT in the field domain. The control circuit for the EPRoC is composed of a 10-MHz bandwidth PLL operated using a reference frequency of 420 MHz (Rohde & Schwarz, SMB100B) and an on-chip 32× divider to obtain a stable oscillation of 13.44 GHz. Frequency-modulated, field-swept CW spectra were acquired using a lock-in amplifier (Anfatec, eLockIn 203) with a modulation frequency of 75 kHz and a peak-to-peak modulation amplitude of 5.3 MHz (0.19 mT) for the a-Si sample, which is equivalent to field modulation when performing resonator-based EPR. For the BDPA spectrum, a modulation amplitude of 0.96 MHz (0.03 mT) and for the mapping 6.4 MHz (0.23 mT) was used. The modulation amplitude for the mapping was deliberately chosen to be about twice the line width to increase the SNR and hence reduce measurement time. The obtained spectra were then linearly baseline corrected and filtered using a second-order Savitzky-Golay filter with a filter window chosen to be small enough that no line broadening was visible.

### Layer structure of EPR-on-a-chip

Figure 1C shows the layer structure of the EPRoC. The coil consists of one Cu and one Al layer that are connected by a Cu via on top of a Si substrate. The coil is passivated with a thin film of SiN and SiO_2_. The topmost layer is a protective polyimide layer (5 μm). The chip was fabricated in a 130-nm CMOS technology (CMRF8SF from GlobalFoundries).

### Mapping apparatus

To determine the sensitive height and volume of the *B*_1_ field in relation to the VCO coils, a custom-designed mapping apparatus was used. A BDPA grain was attached to the tip of a flame-pulled borosilicate capillary with vacuum grease. The pulled capillary was inserted into a Bruker sample holder, which was then attached to a motorized three-axis stage (Thorlabs, PT3-Z8) and suspended above the EPR-on-a-chip board. The three actuators (Thorlabs Z825B and Thorlabs KDC101 controller) have a minimum repeatable incremental movement or step size of 0.2 μm, a backlash of <8 μm, and a bidirectional repeatability of <1.5 μm such that mapping with micrometer precision in *x*, *y,* and *z* directions as indicated in Fig. 1B was possible. The a-Si thin film sample was attached with super glue (UHU Sofortfest) to the opening of a 1.5-mm outer diameter EPR tube that was then inserted in the Bruker sample holder. To align the samples above the chip, two cameras were used. A USB microscope (TOOLCRAFT USB microscope) was used to monitor the *x* and *y* directions, while a DSLM camera (Olympus OM-D E-M5II camera and Olympus M.Zuiko Digital ED 14-150 mm F4-5.6 II lens) was used to observe the *z* direction. For these measurements, the BDPA samples were carefully lowered to the starting position such that the sample would not touch the chip. Therefore, the BDPA experiments were performed in the following order: *z* = 20, 30, 40, 50, 60, and, finally, 10 μm to prevent the sample from being lost when the tip of the flame-pulled capillary comes into contact with the EPRoC. In the experiment, the sample was carefully lowered toward the surface of the EPRoC (i.e., in negative *z* direction) before obtaining the first map. Then, the direction of the movement of the translation stage was reversed (positive *z* direction). Here, the backlash of the translation stage of <8 μm leads to a respective *z* error of the data point at 20 μm. Hence, the relative distance Δ*z* between the data points at 20 and 30 μm may be between 10 and 2 μm, while relative distance between the data points at 30, 40, 50, and 60 μm (Δ*z* ≈ 10 μm) remains viable with an error <1 μm due to the movement of the translation stage in the same direction. For the last data point, the movement direction of the translation stage was reversed (negative *z* direction) a second time. With the backlash, the relative distance between the data point at 60 and 10 μm may be between 50 and 42 μm, leading to the respective *z* error. The thin-film sample, on the other hand, is quite robust and was lowered toward the chip until contacting the surface directly.

### Samples

Two sample classes, namely point-like and thin film samples, were used for the experiments. Single grains of varying sizes of BDPA complex (1:1 with benzene, purchased from Sigma-Aldrich) were used as point-like samples, which were placed on the tip of the pulled capillary described above. BDPA shows an EPR signal at *g* = 2.003 with a line width of about 0.07 mT. The relaxation times of BDPA are about *T*_1_ = 110 ns and $T_{2}^{*}$ = 100 ns (*80*, *81*).

The area of the BDPA samples were estimated by comparison of the sample edge lengths to a printed microscale while viewed under a light microscope. The third edge length was roughly estimated from the shadow present in the micrograph of the sample as was performed in (*61*) and was found to be in the same range (~35 um) as the other two edge lengths. Because the shape of the sample cannot precisely be determined, a cubical sample with an edge length equal to the average of the estimated dimensions was assumed. An error of 10% in the two dimensions that could be readily compared with the printed microscale in the photograph and an error of 50% for the estimation from the shadow present in the sample micrograph were assumed, resulting in a total relative error of 82% for the entire sample volume.

The a-Si was e-beam evaporated in the Von Ardenne CS400PS deposition cluster at PVcomB of HZB (emission current, 560 mA, *T* = 680°C, deposition rate ~ 400 to 450 nm/min, sample rotation 15 rpm) (*85*) on a 5 cm by 5 cm by 500 μm quartz substrate, after which was cut into 1 mm–by–2 mm small pieces using a dicing saw (DISCO DAD3220). The film thickness was 15 μm. The elevated deposition temperature of 680°C avoids the incorporation of hydrogen into the Si layer (*85*). At X- and Q-band frequencies (see fig. S3, A and B), the a-Si exhibits a Voigtian single line EPR signal, which increases in linewidth with increasing frequency as a result of contributions from dangling Si-Si bonds (*78*). The spin density of the sample was determined by means of quantitative EPR with a calibrated spectrometer and is about 2.4(5) × 10^19^ cm^−3^. Because of the high spin density, the relaxation times of the sample could not be determined using pulsed EPR methods. Instead, the relaxation times *T*_1_ and $T_{2}^{*}$ were determined via X-band saturation measurements (see fig. S3C), and the resulting linewidths of the recorded spectra, respectively, are on the order of *T*_1_ ≈ 200 ns and $T_{2}^{*}$ ≈ 20 ns.

### Oscillatory magnetic field simulations

The simulations of the oscillatory magnetic field magnitudes were performed with the finite-element simulation software COMSOL Multiphysics. To reduce computational time, the *B*_1_ field of only one-half coil with the layer structure shown in Fig. 1C on an infinite Si substrate was simulated at 14 GHz with a coil current of 30 mA. To obtain the *B*_1_ distribution of the complete array, the simulated data of this half coil were mirrored, translated relative to the first half coil, superimposed to form a complete coil, and repeated until forming the entire 12-coil array. In this way, the inductive coupling between the coils is not considered. In the plots shown in Fig. 2, the obtained *B*_1_ field was scaled such that the expected coil current matched the value of the bias current corresponding to the experimental settings in the herein described experiments. To calculate the bias current from the coil current, the square-root law proposed in (*61*) was used.

### Simulation of EPR signal dependence on partial utilization of the EPRoC array

The simulation of the FM signal amplitudes when covering a varying number of coils of the injection-locked 12-coil array was performed with SpectreRF Circuit Simulator (Cadence Design Systems) using similar methods as the simulations discussed in (*60*). The simulation is based on the so-called steady-state spin model firstly proposed in (*86*) for NMR and further discussed for EPRoC in (*72*, *87*). In this model, the spin system is assumed to be unsaturated and can therefore be described by an RLC circuit with *R*_spin,sim_, *L*_spin,sim_, and *C*_spin,sim_, which is coupled to the coils of the VCOs 12-coil EPRoC array with a coupling coefficient *K*_spin_ = ηχ_0_. A bias current of 5 mA was used in the simulation, which is equal to that used in the experiments. Please note that the *B*_1_ inhomogeneity was not considered in the simulation, and the *B*_1_ magnitude was calculated at the coil center using Biot-Savart’s law (Eq. 4). The transverse relaxation time, $T_{2}^{*}$, of the a-Si sample of 20 ns was used for the simulation accordingly. The sample volume per coil was calculated from the sensitive volume determined in section EPR signal distribution in a single coil and the thickness of the a-Si film of 15 μm as *V*_s_ = π × 80 × 62.5 × 15 μm^3^ = 2.34 × 10^5^ μm^3^, while the detector volume (*72*) was calculated from the inductance of the coil, *L*_0_, as

$$V_{\text{det}}\approx \frac{\mu_{0}}{B_{1u}^{2}}L_{0}$$

assuming a *B*_1u_ calculated at the coil center to be constant over the sample. From these two values, the filling factor was calculated as η = *V*_s_/*V*_det_. The number of spins per coil was calculated from the spin density of the a-Si sample and *V*_s_, which was used in turn to calculate χ_0_ using Curie’s law. The simulation was performed for a sample present in 1, 3, 6, 9, and 12 coils. For each occupancy, a field-swept FM spectrum of 0th order, i.e., without PSD by the lock-in amplifier, was simulated. From these spectra, the first-order FM spectra, i.e., the first-derivative as obtained with PSD by the lock-in, were numerically calculated considering the modulation amplitude of the experimental data, from which in turn the signal amplitude was determined. A linear increase of the signal amplitude as a function of the number of used coils was found.
