# Supplementary material for: Microwave field mapping for EPR-on-a-chip experiments
Source: Sci Adv. 2024 Aug 16;10(33):eado5467. doi: 10.1126/sciadv.ado5467 (PMC11801239; doi:10.1126/sciadv.ado5467)
Supplement: Supplementary file 1 — Supplementary Text Figs. S1 to S3 [file sciadv.ado5467_sm.pdf]

Science Advances
AAAS

Supplementary Materials for

Microwave field mapping for EPR-on-a-chip experiments

Silvio Künstner *et al.*

Corresponding author: Joseph E. McPeak, joseph.mcpeak@helmholtz-berlin.de

Sci. Adv. **10**, eado5467 (2024)
DOI: 10.1126/sciadv.ado5467

This PDF file includes:

Supplementary Text
Figs. S1 to S3

Supplementary Text

Calculation of the electric field component of the microwave inside a TE-102 cavity resonator

The electric field component of the MW was calculated as follows. Typical dimensions (*x*, *y*, *z*) for a TE-102 cavity resonator were taken from ref. (75): a = 23.5 mm; b = 11.0 mm; d = 42.0 mm. The non-vanishing field components of a TE-102 cavity resonator can be described by (76) (p. 132):

$$H_{1,x} = \frac{H_0}{[1+(d/2a)^2]^{1/2}} \sin\frac{\pi x}{a} \cos\frac{2\pi z}{d} \tag{S1}$$

$$H_{1,z} = \frac{H_0}{[1+(2a/d)^2]^{1/2}} \cos\frac{\pi x}{a} \sin\frac{2\pi z}{d} \tag{S2}$$

$$E_{1,y} = j\left(\frac{\mu_0}{\epsilon_0}\right)^{1/2} H_0 \sin\frac{\pi x}{a} \sin\frac{2\pi z}{d} \tag{S3}$$

where H_0 is the amplitude of the MW magnetic field, μ_0 is the magnetic constant and ε_0 is the electric constant. In the coordinate system in ref. (76), the static magnetic field, B_0, is along z and the sample is inserted along the x-axis at $z = d / 2$. Hence, for the EPR resonance condition, only $H_{1,x}$ is relevant since $H_{1,z} \parallel B_0$. The maximum $H_{1,x}$ is at $x = a / 2$ and $z = d / 2$ (in theory also at $z = 0$ and $z = d$), i.e., in the center of the cavity, where the trigonometric functions are maximal. Assuming a certain $H_{1,x,\mathrm{max}}$ available for EPR, we can rearrange Eq. (S1), to calculate H_0 as

$$H_0 = H_{1,x,\mathrm{max}} \cdot [1+(d/2a)^2]^{1/2}, \tag{S4}$$

which can then be used to calculate $E_{1,y}$, which has its extrema at $x = a / 2$ and $z = d /4$ or $z = 3d / 4$, i.e., about 10.5 mm away from the center of the cavity in the z-direction, and is sinusoidally increasing (from $-E_{1,y}$ at $z = 3d / 4$) or decreasing (from $E_{1,y}$ at $z = d / 4$) to 0 in the center of the cavity at $z = d / 2$ = 21 mm. Assuming a B_1 of 27 μT in the center of the cavity, $H_{1,x,\mathrm{max}} = 2 / \mu_0 B_1$ may be calculated, which is then used to calculate the maximum of $E_{1,y}$, which is therefore approximately 21 kV/m. Please note that the factor of 2 was added to account for the two counter-rotating MW.

Fit of the signal amplitude maps of the 1 coil mapping

Each of the maps of Figure 4A-F was least-square fitted with a 2D Gaussian function as

$$I = b + a \cdot e^{-\frac{(x-x_0)^2}{2c_x^2}-\frac{(y-y_0)^2}{2c_y^2}}, \tag{S5}$$

where the parameters a, b, c_x, c_y, x_0, and y_0 were adjusted. From c_x and c_y, the full width at half maximum (FWHM) may be calculated as $\mathrm{FWHM}_x = 2\sqrt{2\ln 2}c_x$ and $\mathrm{FWHM}_y = 2\sqrt{2\ln 2}c_y$, respectively. Figure S1 shows the resulting fitting parameters. From the FWHM, an asymmetry of the signal amplitude may be seen and is larger in the x-direction than in the y-direction. A similar behavior was observed in the mapping of the 3 coils (Figure 5). Here, however, the spacing between data points in the y-direction was much smaller than in the x-direction, such that the non-uniform spacing may create an elliptical image resulting from truncations in the axis with lower relative spacing. The spacing of data points in the single coil experiment was equal, i.e., $\Delta x = \Delta y$. It is also possible that contributions from the connection of the octagonal coil to the varactor, which exhibits a large B_1 field, may lead to stronger saturation of the sample in this location.

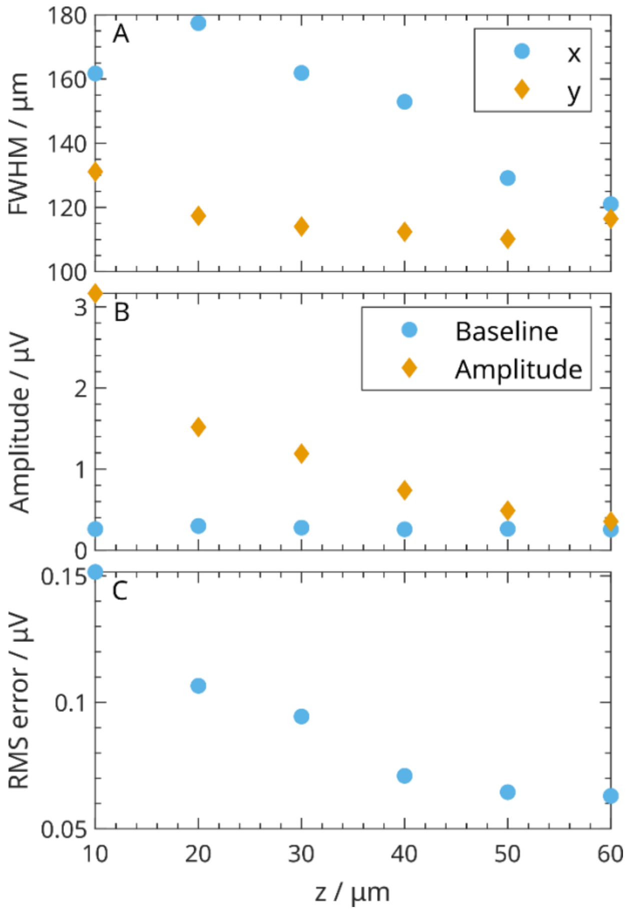

Fig. S1. Parameters of the fit of the 1 coil mapping experiment with a 2D Gaussian. (A) FWHM in *x*- ($FWHM_x$) and *y*-direction ($FWHM_y$). (B) Signal amplitude, *a*, and baseline, *b*. (C) Root mean squared error of the fits.

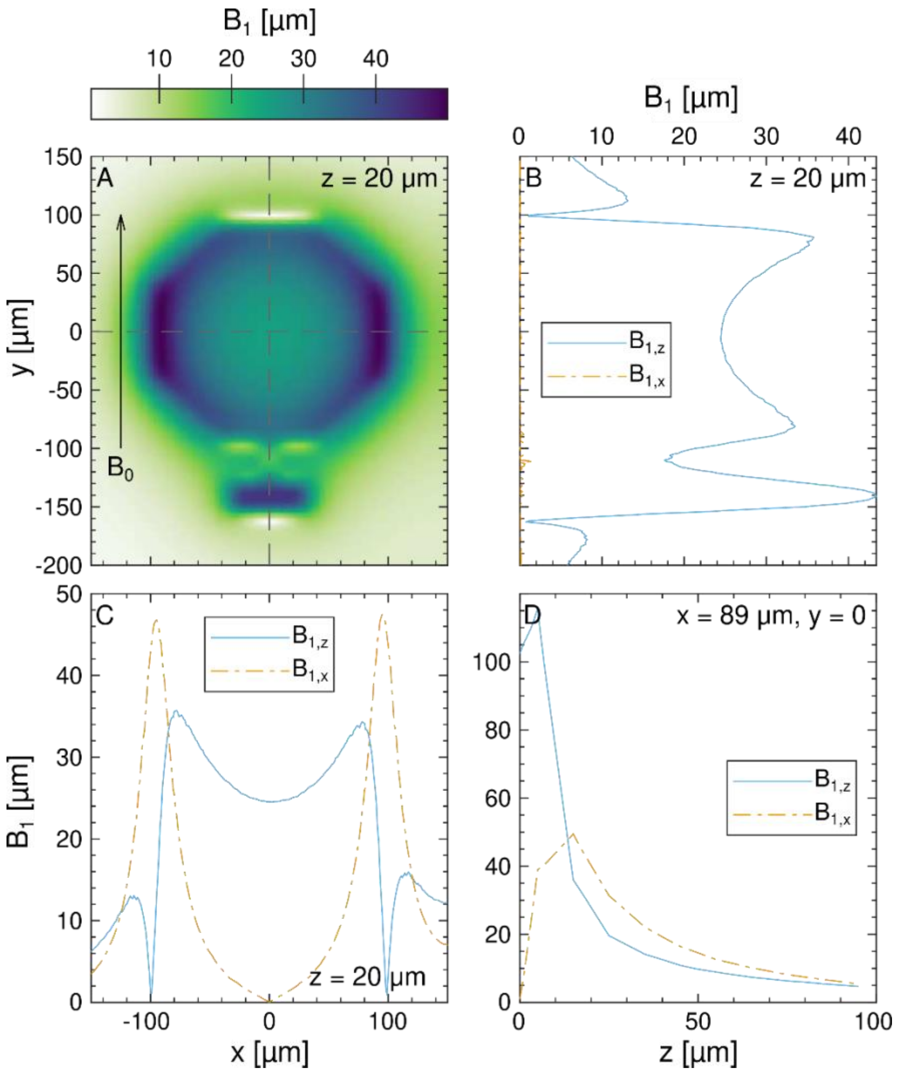

Fig. S2. Simulated distribution of the B_1 field of one coil of the 12-coil EPRoC array using a bias current of 5 mA at a height of 20 µm above the surface of the EPRoC (z = 20 µm). (A) Magnitude of B_1 (⊥ to B_0). As indicated, B_0 is parallel to the y-direction. (B) $B_{1,z}$ and $B_{1,x}$ as a function of y. (C) $B_{1,z}$ and $B_{1,x}$ as a function of x. (D) $B_{1,z}$ and $B_{1,x}$ as a function of z in the center of the coil (x = 89 µm, y = 0).

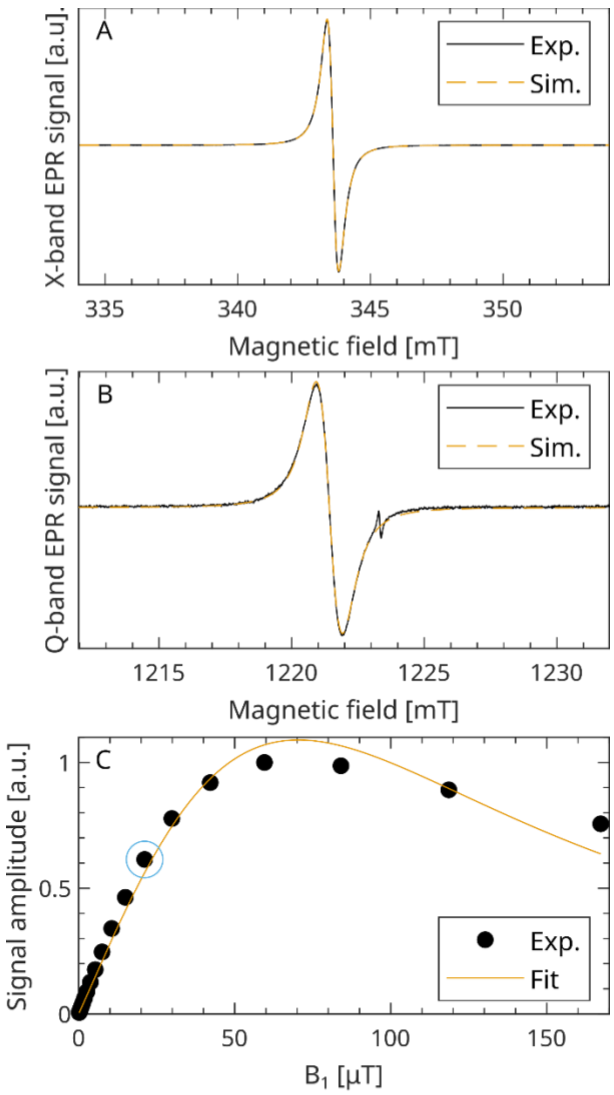

Fig. S3. Resonator-based EPR data of the a-Si thin film sample. (A) X-band spectrum and simulation. (B) Q-band spectrum and simulation. The second line with much lower signal intensity right of the a-Si line is the quartz glass substrate (C) Saturation behavior in the X-band displayed as the peak-to-peak signal amplitude as a function of the magnitude of the magnetic field component of the MW, B_1, calculated from the resonator conversion factor and the applied MW power. The relaxation times of the sample were determined from the peak-to-peak linewidth of the X-band spectrum and the fit of the saturation curve. The open circle depicts the B_1-value used to acquire the X-band spectrum.
